# Identification of Genome Sequences of Polyphosphate-Accumulating Organisms by Machine Learning

**DOI:** 10.3389/fcell.2020.626221

**Published:** 2021-01-18

**Authors:** Bohan Liu, Jun Nan, Xuehui Zu, Xinhui Zhang, Qiliang Xiao

**Affiliations:** State Key Laboratory of Urban Water Resource and Environment, School of Environment, Harbin Institute of Technology, Harbin, China

**Keywords:** polyphosphate-accumulating organisms, genome sequences, minimap2, support vector machine, prediction

## Abstract

In the field of sewage treatment, the identification of polyphosphate-accumulating organisms (PAOs) usually relies on biological experiments. However, biological experiments are not only complicated and time-consuming, but also costly. In recent years, machine learning has been widely used in many fields, but it is seldom used in the water treatment. The present work presented a high accuracy support vector machine (SVM) algorithm to realize the rapid identification and prediction of PAOs. We obtained 6,318 genome sequences of microorganisms from the publicly available microbial genome database for comparative analysis (MBGD). Minimap2 was used to compare the genomes of the obtained microorganisms in pairs, and read the overlap. The SVM model was established using the similarity of the genome sequences. In this SVM model, the average accuracy is 0.9628 ± 0.019 with 10-fold cross-validation. By predicting 2,652 microorganisms, 22 potential PAOs were obtained. Through the analysis of the predicted potential PAOs, most of them could be indirectly verified their phosphorus removal characteristics from previous reports. The SVM model we built shows high prediction accuracy and good stability.

## Introduction

Phosphorus (P) is one of the key element controlling the normal functioning of many organisms in the ecosystem (Zeng et al., 2004). In aqueous environment, phosphorus compounds could be hydrolyzed to orthophosphate, the only form of phosphorus which could be utilized by aquatic organisms (Correll, 1998). However, presence of excess phosphate into the water bodies, carried by domestic and industrial wastewater, may trigger eutrophication, which lead to algae bloom, oxygen depletion in water, and biological organism death (Smith et al., 1999; Ju et al., 2016). Study shows that about 260,000 metric tons of P were discharged from wastewater treatment facilities every year in the US (Litke, 1999; Loganathan et al., 2014). With the significant increase of phosphorus levels in the water environment, people are concerned about the deterioration of water quality and overall ecological balance (Stoddard et al., 2016).

Among of all methodologies, biological phosphorus removal (BPR), as the most economical strategy was widely used in wastewater treatment plants (WWTP) (Huang et al., 2020). Previous studies suggested that polyphosphate-accumulating organisms (PAOs) are able to remove P removal from BPR system (Cai et al., 2019; Gao et al., 2019; Wang et al., 2019). It is mainly because of the special metabolism of PAOs under alternative anaerobic and aerobic reaction. Under the anaerobic reaction, the polyP stored in PAOs are degraded to orthoP and releases into the liquid with the orthoP adsorbed by EPS. Under the aerobic reaction, a part of orthoP passes through EPS and stores P as polyP inside PAOs, and the other part can be adsorbed by the surrounding EPS matrix (Liu et al., 2020). One of such study reported that over 90% P was removed under aerobic environment in a sequencing batch reactor by PAOs. Therefore, it is feasible to increase the content of PAOs in the activated sludge to improve the P removal efficiency. The pre-requisite for increasing the content of PAOs is to be able to effectively identify PAOs. Recently, some work has been reported in the identification of PAOs, just like Dechloromonas (Kong et al., 2007; Günther et al., 2009), *Candidatus accumulimonas* spp. (Nguyen et al., 2012), *Microlunatus* spp. (Kawaharasaki et al., 1999; Beer et al., 2004), *Aeromonas* (Wang et al., 2009), *Tetrasphaera* spp. (Kong et al., 2005; Nguyen et al., 2011), *Pseudomonas* (Shi and Lee, 2006), *Klebsiella* (Sun et al., 2016), *Rhodopseudomonas* (Tsuneda et al., 2005), and so on. However, due to the variety of PAOs, although more than 7,000 species have been identified, there are still many strains of PAOs that have not been found. At present, most of the existing researches on PAOs were based on gene-level analysis. The species and the relative abundance of PAOs of biological phosphorus removal system could be investigated by high-throughput sequencing technology (Lin et al., 2019; Salehi et al., 2019; Xu et al., 2019). However, it is cumbersome and time-consuming to sieve and identify the PAOs by conventional experimental methods. Therefore, it is particularly important to find a fast and effective method to identify the PAOs.

Recently, algorithms have been widely used in medicine, geology, hydrology and so on, due to its convenience, quickness and accurate prediction (Fijani et al., 2018; Pham et al., 2018; Peng and Zhao, 2020; Zhao et al., 2020a,b,c,d). Pang et al. (2019) developed a fluctuant influent responsive Q-learning based BPR optimizing control method. An efficient approach based on bi-sensitivity analysis and genetic algorithm for calibration of activated sludge models was proposed by Chen et al. (2015). However, the algorithm for the identification of PAOs has not been reported yet. Identification of PAOs, which is described as a classification problem, can be achieved using supervised classification algorithms. The support vector machine (SVM) is an effective and intelligent machine learning method which can solve classification problems and non-linear function estimation problems in the fields of pattern recognition and machine learning (Vapnik, 1995; Chen et al., 2008; Cheng and Jhan, 2012). In the context of intelligent classification, SVM combined with feature extraction method has been successfully employed in the field of pattern recognition (Ballabio and Sterlacchini, 2012; Chen et al., 2017; Shi et al., 2017; Fijani et al., 2018; Fu et al., 2019). Besides, under the same detection performance conditions, SVM require less prior knowledge than other methods, and the training time can be greatly shortened, which provides favorable conditions for the identification of genome sequences. To the best of our knowledge, there are few studies till now applying SVM to distinguish and identify PAOs in the complex biological activated sludge.

In this study, a SVM algorithm was proposed to realize the rapid identification of PAOs in activated sludge system. The genome sequences alignment of thousands of bacterial groups were used to find out the characteristic genome of PAOs. The main objective of this study was to establish a new model through the SVM algorithm, which can use the features of the genome sequences to achieve large-scale and high-precision identification of PAOs, with an accuracy of over 90%. This development illustrated a novel and efficient strategy to accelerate the screening and identification of microorganism.

## Materials and Methods

### Method Overview

**Figure 1:**
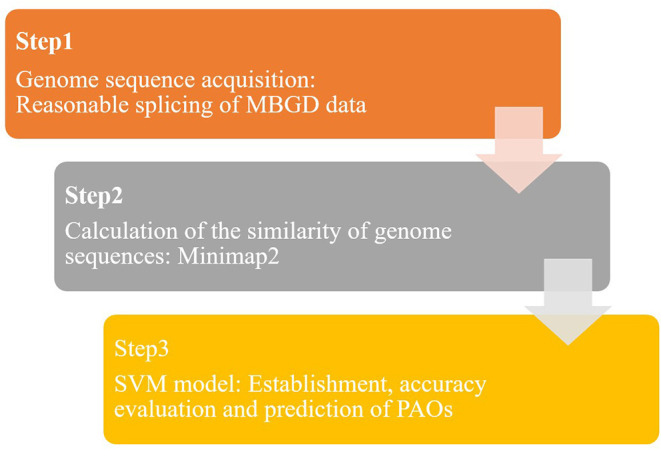
The framework of the identification of genome sequences of PAOs.

In this study, a predictive model of PAOs was established based on the SVM algorithm, to realize rapid and accurate identification of PAOs. The SVM-based identification method of PAOs was divided into three steps (Figure 1): genome sequences collection, pairwise alignment for genome sequences and the identification of PAOs based on SVM.

Step 1: The genome sequences of microorganisms were extracted from the publicly available microbial genome database for comparative analysis (MBGD). New long-chain genome sequences were obtained by splicing the genome sequences in MBGD database to serve as training databases.

Step 2: Data processing was conducted to get a series of s the similarity between genome sequences, based on the database formed from MBGD (Step 1). Minimap2 was used to compare the obtained genome sequences in pairs, and read the overlap. By calculating the overlap, the similarity between genome sequences was achieved.

Step 3: The SVM model based on the prediction of PAOs was established. Through feature selection of the above similarity, we got a matrix of 1^*^6,318. The accuracy of the SVM model is verified by grouping the microorganisms reasonably. In addition, over 2,000 species of bacteria were identified based on this SVM model to predict the potential PAOs.

### Genome Sequences Collection

For this study, all the genome sequence datasets were retrieved from the publicly available microbial genome database for comparative analysis (MBGD, available at http://mbgd.genome.ad.jp/). As its unique feature, MBGD allows users to perform orthology analysis among any specified set of organisms (Uchiyama et al., 2013). It is this flexibility that makes MBGD adapt to a variety of microbial genomic study. Due to the huge diversity of microorganisms, a total of 6,318 representative microbial genome sequences including 5,861 bacteria, 254 archaea and 203 eukaryota stored in the MBGD were used for analysis. However, the gene sequence of each microorganism provided by MBGD is divided into several thousand groups that cannot be directly compared. Therefore, these genome sequences were spliced to reconstitute a new genome sequence for similarity comparison.

### Similarity Comparison of Genome Sequences

**Figure 2:**
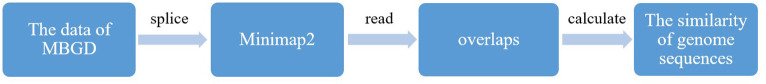
Calculation of the similarity of the genome sequences by minimap2.

By splicing the genome sequences of microorganisms provided by the MBGD, each microbe has obtained a long-chain gene. In this study, we used minimap2 to rapidly obtain all-to-all read overlaps (Figure 2). Minimap2 was a first DNA and RNA-seq aligner specifically designed for long sequence alignment (Li, 2018). In terms of long sequence alignment, minimap2 achieved approximate mapping 50 times faster than BWA-MEM (Li, 2016). Minimap2 is based on identifying reads that share many co-linear minimizers (Roberts et al., 2004). Minimap2 uses indexing and seeding algorithms similar to minimap (Li, 2016), and furthers the predecessor with more accurate chaining, the ability to produce base-level alignment and the support of spliced alignment. We used minimap2 to compare the genomes of the obtained microorganisms in pairs, and read the overlap. By calculating the overlaps, the similarity between the microbial genome sequences were available.

### Support Vector Machine (SVM)

The support vector machine (SVM) is one of the most successful machine learning algorithm in the prediction field developed by Vapnik (1995). It has been widely applied in bioinformatics, computational biology and environmental studies (Chen et al., 2012; Liu and Lu, 2014; Ding et al., 2016; Pan et al., 2018). The SVM model is used to find the optimal solution separating two classes which adopts the theory of structural risk minimization instead of empirical risk minimization to reduce the over-fitting problem. Therefore, the SVM algorithm is very effective for the identification and prediction of PAOs. In addition, unlike traditional machine learning, SVM can convert the complex non-linear problem input data to a higher-dimensional space where a hyperplane is constructed by introducing kernel function (Vapnik and Vapnik, 1998; Carrier et al., 2013; Ch et al., 2013; He et al., 2014). The main idea of SVM is to create a line or a hyperplane as the decision surface that maximizes the margin between two classes. The hyperplane function can be defined as follows:

(1) $$\begin{array}{r} w^{T}x+b=0 \end{array}$$

where *w* represents the weight vector, *b* is the bias parameter, and *x* indicates the input vector in the sample space. Taking binary classification problem as an example, to correctly classify samples, all samples are required to meet the following constraints:

(2) $$\begin{array}{r} w^{T}x_{i}+b\left\{ \begin{array}{c} >1\;y_{i}=1\\ <-1\;y_{i}=-1 \end{array}\right. \end{array}$$

However, in practice, some abnormal points that cannot be linearly separated often exist. In order to solve this problem, a slack variable ξ_*i*_ and penalty factor *C* are added. The optimization function and constraints are as follows:

(3) $$\begin{array}{r} Min\left(\frac{1}{2}\|w{\|}^{2}+C\overset{n}{\underset{i=1}{\sum }}\xi_{i}\right)\\ s.t.,\left\{ \begin{array}{c} y_{i}\left(wx_{i}+b\right)\geq 1-\xi_{i}\\ \xi >0 \end{array}\right. \end{array}$$

For simplifying the calculation, the above equation can be transformed into solving the saddle point of the Lagrange equation by using the Lagrange multiplier. By applying the duality theorem, the final optimization problem is presented as:

(4) $$\begin{array}{r} Max\left\{\overset{n}{\underset{i=1}{\sum }}\lambda_{i}-\frac{1}{2}\;\overset{n}{\underset{i=1}{\sum }}\overset{n}{\underset{j=1}{\sum }}\lambda_{i}\lambda_{j}y_{i}y_{j}\left(x_{i}\;x_{j}\right)\right\}\\ s.t.,\;\overset{n}{\underset{i=1}{\sum }}\lambda_{i}y_{i}=0,\;\alpha_{i}\geq 0\;\left(4\right) \end{array}$$

where λ_*i*_ is Lagrange multiplier.

This problem can be solved by using quadratic programming. The final linear discriminant function that is used for the classification of new data can be achieved as follows:

(5) $$\begin{array}{r} f\left(x\right)=sgn\left(\overset{n}{\underset{n=1}{\sum }}\lambda_{i}y_{i}\left(xx_{i}\right)+b\right) \end{array}$$

However, after mapping the original space into the higher dimensional feature space, different inner product kernel functions will form different algorithms. As we know, four types of kernel functions are available in SVM including polynomial kernel (PL), sigmoid kernel (SIG), radial basis function (RBF) and linear kernel (LN). Besides, the prediction accuracy would be also enhanced with appropriate kernel function. In the previous stage, we have performed a pairwise comparison of the gene sequences of the 6,318 strains in MBGD. By calculating the similarity, we get a matrix of 1^*^6318. Since the feature dimension we got is already very high, there is no need to perform higher-dimensional mapping by other kernel functions. Therefore, in this method, we employed the LN function to train our predictive models, which is defined as:

(6) $$\begin{array}{r} K\left(x_{i},x_{j}\right)=x_{i}x_{j} \end{array}$$

The kernel function is used to transform data into two classes consisting of unknown bacteria and PAOs {0, 1}.

The optimal classification function can be constructed as follows:

(7) $$\begin{array}{r} f\left(x\right)=sgn\left(\overset{n}{\underset{n=1}{\sum }}\lambda_{i}y_{i}K\left(x_{i},x\right)+b\right) \end{array}$$

## Results

### Data Pre-processing

Experiments adopted an MBGD data which combined the Reference Sequences (RefSeq) database of National Center for Biotechnology Information (NCBI), the original GenBank entry, and the Gene Trek in Prokaryote Space (GTPS) provided by DNA Data Bank of Japan (DDBJ). The genome sequences of 6,318 microorganisms were provided by MBGD. However, the genome sequence of each microorganism provided by MBGD is divided into several thousand groups that cannot be directly compared. Therefore, we made a reasonable splicing of the microbial genome sequences, so that each microbial obtained a long chain of genome sequence. Feature selection was used before SVM algorithm for choosing a subset of the features that is clean of redundancies. Moreover, it is necessary to select highly correlated features over other features to improve the prediction accuracy in the SVM model. After obtaining the microbial genome sequences, minimap2 which is a general-purpose alignment program to map DNA or long mRNA sequences against a large reference database was used to compare the genome sequences, and read the overlap. By calculating the overlaps, the similarity between the microbial genome sequences were available. We obtained a matrix of 1^*^6318 to complete the feature selection.

### SVM Experiments

**Figure 3:**
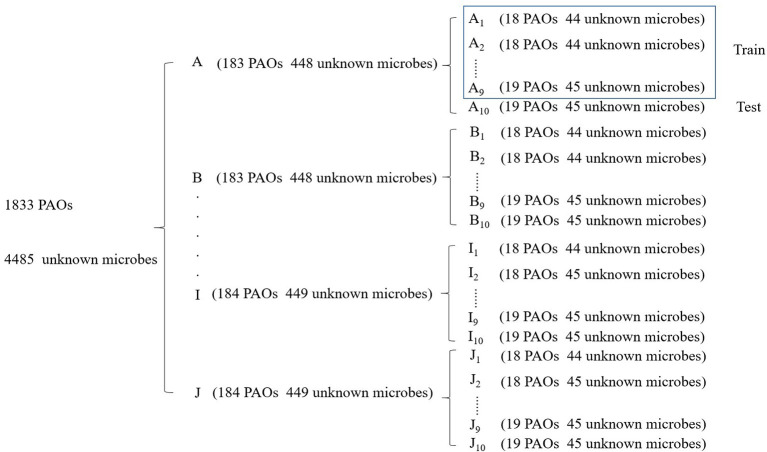
Ten-fold cross-validation of the SVM model.

**Figure 4:**
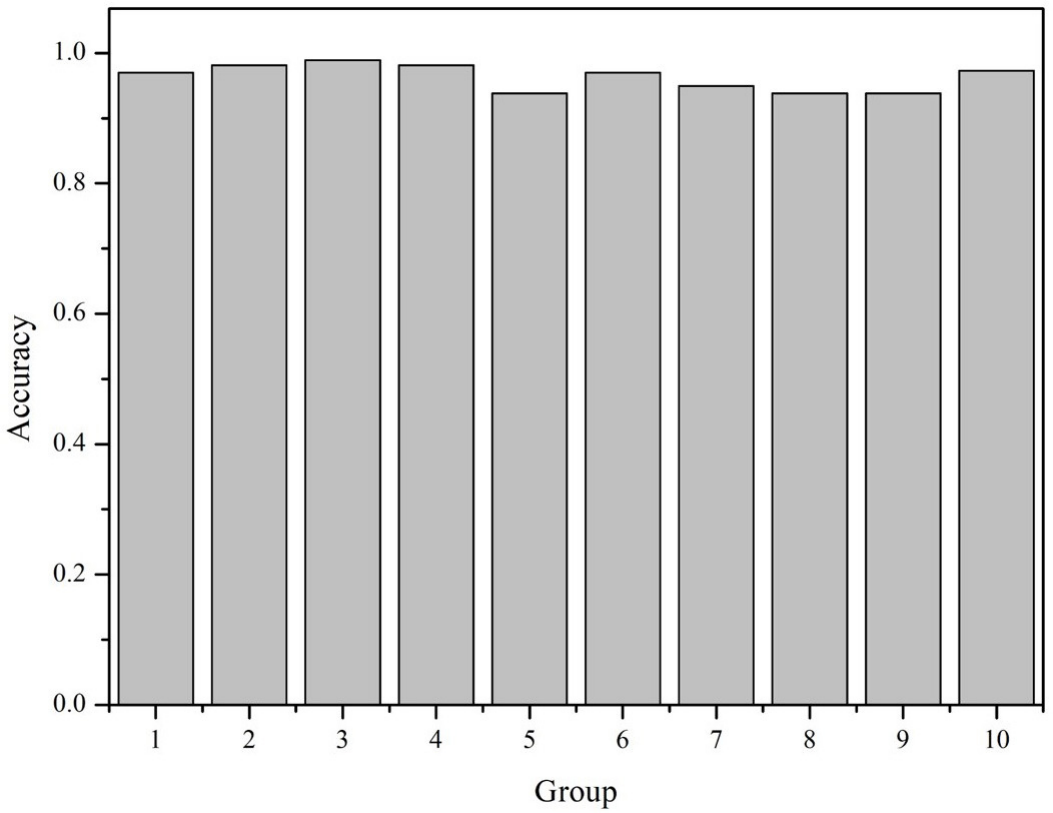
The accuracy of the SVM model.

All the 6,318 microbial genome sequences were provided by MBGD, including 1,833 known PAOs, such as Dechloromonas, Pseudomonas, Rhodopseudomonas, and Klebsiella. The remaining 4,485 microbes have not been proved whether they are PAOs. All the 1,833 known PAOs and the remaining 4,485 microbes were selected to form the sample database of the SVM model. All the data were classified into [0, 1], 0 for the microorganisms that are uncertain whether they are PAOs, and one for the PAOs. Subsequently, the features obtained were trained by the SVM classifier, which were then evaluated with 10-fold cross-validation to test the accuracy of the algorithm (Figure 3). 10-fold cross-validation is a common test method which is to divide the sample database into ten parts, and takes turns using nine parts as training data and one part as test data for testing. The corresponding accuracy rate can be obtained in each experiment, and the average accuracy of the 10 results can be used to evaluate the accuracy of the algorithm. In the SVM model, the 1,833 known PAOs and the remaining 4,485 microbes were divided into 10 parts with 183~184 PAOs and 448~449 unknown microbes randomly. In order to improve the accuracy of cross-validation, the above 10 parts of data were divided into 10 groups randomly. Each group contained 18~19 PAOs and 44~45 unknown microbes. For the 10 groups of data in each part, we randomly selected nine groups of data to train the SVM model, and the remaining one group of data for testing. In other words, of all the data, 90% were used for training and 10% were used for testing. The Figure 4 illustrated the satisfactory prediction accuracy of the SVM models in the testing stage. The test accuracy of the SVM model for 10 parts of data are: 0.9700, 0.9810, 0.9889, 0.9810, 0.9383, 0.9699, 0.9493, 0.9382, 0.9382, and 0.9731. The average accuracy provided by the SVM model is 0.9628±0.019. Therefore, the SVM model shows high prediction accuracy and good stability, and can be used for the prediction of PAOs in the future.

## Discussion

**Figure 5:**
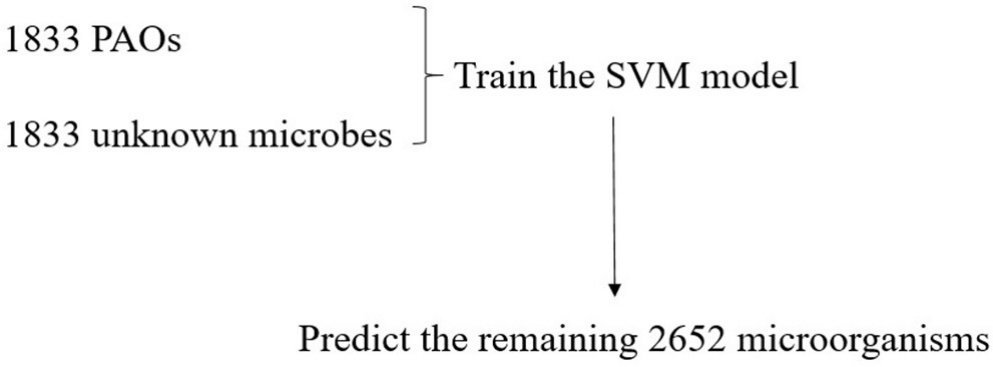
The prediction of the PAOs by the SVM model.

Due to the high accuracy and good stability of the SVM model, it can be used to predict PAOs. In this experiment, we selected 1,833 of all known PAOs, and randomly selected 1,833 from the remaining microorganisms as the negative samples to test the model. Subsequently, we made predictions on the remaining 2,652 microorganisms and identified potential PAOs that may not be discovered (Figure 5).

It can be seen from the prediction results that we have predicted 22 potential PAOs, namely: *Anoxybacillus flavithermus* WK1, *Arcanobacterium haemolyticum* DSM 20,595, *Anoxybacillus* sp. B7M1 b7m1, *Anoxybacillus* sp. B2M1 b2m1, *Arthrobacter phenanthrenivorans* Sphe 3, *Amphilbacillus xylanus* NBRC 15112, *Brevibacillus brevis* NBRC NBRC 100599, *Corallococcus coralloides* DSM 2259, *Arthrospira* sp. PCC 8005, *Salmonella enterica* subsp. Enterica serovar Choleraesuis SCSA50, *Campylobacter jejuni* subsp. jejuni RM3196, *Pannonibacter phragmitetus* 31801, *Helicobacter pylori* ML1, *Shigella flexneri* 2a 981, *Lactobacillus plantarum* JBE245, *Sulcia muelleri* PUNC, *Jannaschia* sp. CCS1, *Kinetoplastibacterium oncopeltii* TCC290E, *Thiomonas* sp. 3As, *Teredinibacter turnerae* T7901, *Wolbachia endosymbiont* TRS, *Wolbachia endosymbiont* of *Drosophila simulans* wHa. All the above 22 kinds of microorganisms are potential PAOs which have not been reported, and their characteristics are analyzed as follows.

*A. flavithermus* WK1 was isolated from the waste water drain at the Wairakei geothermal power station in New Zealand (Mountain et al., 2003). It is a member of the family Bacillaceae. *A. flavithermus* grow only at ~30~72°C (Heinen et al., 1982), which are always found in high-temperature habitats such as geothermal hot springs, the manufacture of milk powder, and manure (Heinen et al., 1982; Pikuta et al., 2000; Clerck et al., 2004; Rueckert et al., 2005). So, it is a typical thermophilic bacteria strain. Activated sludge process is generally conducted under the conditions of 12 °C~25 °C, which is not conducive to the growth of *A. flavithermus* WK1. Therefore, it has not been reported that *A. flavithermus* WK1 is PAOs. *A. haemolyticum* DSM 20595, a Gram-positive bacterium, can cause a wide range of diseases in humans, just as wound infections and pharyngitis (Banck and Nyman, 1986; Miller et al., 1986; Mackenzie et al., 1995; Linder, 1997). *A. haemolyticum* was previously in the Corynebacterium genus, but it is classified as a member of Actinobacteria now (Collins et al., 1982). It has been reported that most of the Actinobacteria may be PAOs, so we conclude that *A. haemolyticum* DSM 20595 is most likely to be a potential PAOs. Both *Anoxybacillus* sp. B7M1 b7m1 and *Anoxybacillus* sp. B2M1 b2m1 belong to *Anoxybacillus* which is closely related to the genus *Geobacillus* and the member of the family Bacillaceae (Pikuta et al., 2000). All of them are Gram-positive bacterium and thermophiles with optimum growth temperature around 55 °Cwhich are similar to the *A. flavithermus* WK1 (Goh et al., 2013). Besides, members of *Anoxybacillus* have been proved great potential in environmental applications (Zitomer et al., 2007; Ghaffari et al., 2011). Therefore, *Anoxybacillus* sp. B7M1 b7m1 and *Anoxybacillus* sp. B2M1 b2m1 are likely to have a special capture effect on phosphate. *Amphibacillus xylanus* strains were previously isolated from an alkaline compost. The genome of *A. xylanus* NBRC 15112 (GenBank: AP012050.1; Taxonomy ID: 698758) was released in 2013, and the type strain was confirmed to utilize xylan from oat spelt and larchwood, and to grow between pH 8 and pH 10 (Niimura et al., 1990). *Amphibacillus* is a member of Bacillus which is considered as PAOs. Therefore, *A. xylanus* NBRC 15112 is also likely to be a potential PAOs. *A. phenanthrenivorans* Sphe 3 is a Gram-positive, aerobic, novel type strain of the genus Arthrobacter belonging to Actinobacteria which was isolated from a creosote-contaminated soil in Epirus, Greece (Kallimanis et al., 2007). It can grow on phenanthrene as the sole source of carbon and energy with a suitable temperature of 30 °C~37 °C and pH of 7.0~7.5 (Kallimanis et al., 2011). Just like *A. haemolyticum* DSM 20595, due to belong to Actinobacteria, it is most likely an undiscovered PAOs. *B. brevis* NBRC NBRC 100599 is a Gram-positive and spore-forming bacterium which has a broad-spectrum antimicrobial activity by secreting some functional metabolites (Song et al., 2012; Pawlowski et al., 2016). Besides, some report indicated that *B. brevis* might be an effective strain for the degradation of polycyclic aromatic hydrocarbons (Zhu et al., 2019). However, whether it also has effect of P removal is not yet known. A potentially novel energy taxis cluster was found in *C. coralloides* DSM 2259 that has been proved to be the same as Actinobacteria. Amongst 34 sequenced myxobacterial genomes, *C. coralloides* is the only species that encodes such a CSS cluster (Huntley et al., 2012). Maybe this cluster of genes gives it the characteristics of PAOs. *P. phragmitetus* 31801 was isolated from the blood sample of a patient with liver abscess (Wang et al., 2017). Currently, the studies on *P. phragmitetus* mainly focused on its bioremediation potentials including reduction of heavy metal chromium and detoxification of polycyclic aromatic compounds (PAHs) under extreme conditions (Borsodi et al., 2005; Xu et al., 2011; Shi et al., 2012; Wang et al., 2013). *Jannaschia* sp. strain CCS1is an ecologically relevant marine proteobacterium found in coastal and open surface waters (Bakolitsa et al., 2010). However, whether they can remove phosphate is not yet known. *Arthrospira* sp. PCC 8005, better known as spirulina, provides exceptional nutritional value which was selected by the European Space Agency (ESA) for its nutritive value and oxygenic properties in the Micro-Ecological Life Support System Alternative (MELiSSA) life support system. It is a photosynthetic prokaryote that plays a crucial role in the Earth's nitrogen and phosphorus cycles. Due to the unique photosynthesis, it has a certain absorption effect on phosphorus (Janssen et al., 2010; Deschoenmaeker et al., 2014). *S. enterica* subsp. Enterica serovar Choleraesuis SCSA50, *C. jejuni* subsp. jejuni RM3196 and *S. flexneri* 2a 981 can cause severe invasive disease in humans just like food-borne bacterial gastroenteritis and diarrheal disease (Hughes and Cornblath, 2005; Peng et al., 2009; Senior, 2009; Parker et al., 2015). The infection of these pathogens are caused by the consumption of contaminated food or drink in humans and animals. Similarly, *H. pylori* ML1 is associated with chronic active type B gastritis and peptic ulcer diseases (Chaun, 2001). These pathogens are not recommended for use in the field of water treatment, so whether they have the effect of removing phosphate is not yet known. *L. plantarum* JBE245 is a member of *L. plantarum* which is isolated from malolactic fermentation of apple juice (Heo and Uhm, 2017). Some work reported that *L. plantarum* strains possess organophosphorus pesticide-degrading activity (Li et al., 2018). Therefore, *L. plantarum* JBE245 was probably an undiscovered PAOs. *S. muelleri* PUNC, *T. turnerae* T7901, *K. oncopeltii* TCC290E, *W. endosymbiont* TRS, and *W. endosymbiont* of *D. simulans* wHa are as intracellular endosymbionts found in specialized cells in organisms (James and Ballard, 2000; Moran et al., 2005; Yang et al., 2009; Alves et al., 2013). They are very likely to ingest phosphate from microbes to maintain the growth. However, these endosymbionts are difficult to appear in the activated sludge. Representatives of the Thiomonas genus are commonly found in moderately acidic (pH 3~5) mine drainage waters (Hallberg and Johnson, 2003; Battaglia-Brunet et al., 2006; Duquesne et al., 2008). Thiomonas strain 3As was isolated from a stream draining an abandoned lead zinc silver mine in the south of France (Duquesne et al., 2008). Like Tm. arsenivorans and Tm. intermediaT (Battaglia-Brunet et al., 2006), strain 3AsT can oxidize As(III) as well as thiosulfate at low pH (Duquesne et al., 2008). In view of the apparently ambiguous available phylogenetic and physiological information regarding strain 3As, whether it has the ability of removing phosphate is not yet known.

The following information can be obtained by analyzing the predicted PAOs. Although Anoxybacillus and Amphibacillus are the members of Bacillus which is considered as PAOs, they are classified separately in MBGD. Through the recognition of *A. flavithermus* WK1, *Anoxybacillus* sp. B7M1 b7m1, *Anoxybacillus* sp. B2M1 b2m1, and *A. xylanus* NBRC 15112, the accuracy of SVM model is proved indirectly. There are also some microorganisms, just like *A. haemolyticum* DSM 20595, *A. phenanthrenivorans* Sphe 3, and *C. coralloides* DSM 2259, belonging to Actinobacteria or part of gene sequences are the same as Actinobacteria. Some work have been reported that most of the Actinobacteria may be PAOs. Some others are the endosymbionts ingesting P from the hosts to maintain the growth, but not common in activated sludge. It is worth noting that *L. plantarum* JBE245, belonging to *L. plantarum*, has the degradation characteristics for organophosphorus. All of them have been predicted by SVM model which can be seen in the Table 1, and the accuracy of the model is proved.

**Table 1:** Comparison of the predicted PAOs.

**Predicted PAOs**	**Result**
*A. flavithermus* WK1	Potential PAOs
*A. haemolyticum* DSM 20595	Potential PAOs
*Anoxybacillus* sp. B7M1 b7m1	Potential PAOs
*Anoxybacillus* sp. B2M1 b2m1	Potential PAOs
*A. phenanthrenivorans* Sphe 3	Potential PAOs
*A. xylanus* NBRC 15112	Potential PAOs
*B. brevis* NBRC NBRC 100599	Unknown
*C. coralloides* DSM 2259	Potential PAOs
*Arthrospira* sp. PCC 8005	Potential PAOs
*S. enterica* subsp. Enterica serovar Choleraesuis SCSA50	Unknown
*C. jejuni* subsp. jejuni RM3196	Unknown
*P. phragmitetus* 31801	Unknown
*H. pylori* ML1	Unknown
*S. flexneri* 2a 981	Unknown
*L. plantarum* JBE245	Potential PAOs
*S. muelleri* PUNC	Potential PAOs
*Jannaschia* sp. CCS1	Unknown
*K. oncopeltii* TCC290E	Potential PAOs
*Thiomonas* sp. 3As	Unknown
*T. turnerae* T7901	Potential PAOs
*W. endosymbiont* TRS	Potential PAOs
*W. endosymbiont* of *D. simulans* wHa	Potential PAOs

## Conclusion

The present work presented a high accuracy SVM algorithm to realize the rapid identification and prediction of PAOs. In this work, all the 6,318 microbial genome sequences were obtained from MBGD. Before the SVM model was established, minimap2 was used to compare the genome sequences. By calculating the similarity between the microbial genome sequences, a matrix of 1^*^6,318 was achieved. The features were trained by the SVM model, and then they were evaluated with 10-fold cross-validation. The average accuracy we got is 0.9628 ± 0.019. Therefore, the SVM model shows high prediction accuracy and good stability. By predicting 2,652 microorganisms, 22 potential PAOs were obtained. Through the analysis of the predicted potential PAOs, most of them could be indirectly verified their phosphorus removal characteristics from previous reports. This result also further confirmed the accuracy of the model we built. This development illustrated a novel and efficient method to accelerate the screening and identification of microorganism. We also look forward to expanding this work to predict other functional bacteria to provide more possibilities for water treatment.

## Data Availability Statement

Publicly available datasets were analyzed in this study. This data can be found here: http://mbgd.genome.ad.jp.

## Author Contributions

BL wrote the paper and did the experiments. JN provided ideas of this work and supervised this work. XZu revised this manuscript. XZh researched the literature. QX organized the data. All authors contributed to the article and approved the submitted version.

## Conflict of Interest

The authors declare that the research was conducted in the absence of any commercial or financial relationships that could be construed as a potential conflict of interest.
